# Drug repositioning or target repositioning: A structural perspective of drug-target-indication relationship for available repurposed drugs

**DOI:** 10.1016/j.csbj.2020.04.004

**Published:** 2020-04-13

**Authors:** Daniele Parisi, Melissa F. Adasme, Anastasia Sveshnikova, Sarah Naomi Bolz, Yves Moreau, Michael Schroeder

**Affiliations:** a ESAT-STADIUS, KU Leuven, B-3001 Heverlee, Belgium; b Biotechnology Center (BIOTEC), Technische Universität Dresden, 01307 Dresden, Germany

**Keywords:** Drug repositioning, Drug repurposing, Drug-target interaction prediction, Drug-target-indication relationship, Structure-based drug repositioning

## Abstract

Drug repositioning aims to find new indications for existing drugs in order to reduce drug development cost and time. Currently,there are numerous stories of successful drug repositioning that have been reported and many repurposed drugs are already available on the market. Although drug repositioning is often a product of serendipity, repositioning opportunities can be uncovered systematically. There are three systematic approaches to drug repositioning: disease-centric approach, target-centric and drug-centric. Disease-centric approaches identify close relationships between an old and a new indication. A target-centric approach links a known target and its established drug to a new indication. Lastly, a drug-centric approach connects a known drug to a new target and its associated indication. These three approaches differ in their potential and their limitations, but above all else, in the required start information and computing power. This raises the question of which approach prevails in current drug discovery and what that implies for future developments. To address this question, we systematically evaluated over 100 drugs, 200 target structures and over 300 indications from the Drug Repositioning Database. Each analyzed case was classified as one of the three repositioning approaches. For the majority of cases (more than 60%) the disease-centric definition was assigned. Almost 30% of the cases were classified as target-centric and less than 10% as drug-centric approaches. We concluded that, despite the use of umbrella term “drug” repositioning, disease- and target-centric approaches have dominated the field until now. We propose the use of drug-centric approaches while discussing reasons, such as structure-based repositioning techniques, to exploit the full potential of drug-target-disease connections.

## 1 Introduction

### 1.1 Drug repositioning to tackle pharmaceutical R&D decline

Drug discovery is a complex and challenging process with an estimated success rate of only 2% [1]. Such a high rate of failure raises the average cost of drug discovery to US $2–3 billion [2]. However, it is sometimes possible to use approved drugs or investigational molecules to treat conditions that differ from the intended purpose. Sildenafil is a well-known example that was first developed to treat hypertension but was eventually commercialized for the treatment of erectile dysfunction [3]. The story of dimethyl fumarate, which was used in Europe for over 20 years in the treatment of psoriasis [4], represents another interesting example of drug repositioning. Only recently has dimethyl fumarate been re-discovered and in 2013 approved to treat multiple sclerosis [5]. Even undesired effects of a drug can be beneficial in the context of another indication. In the tragic case of thalidomide, its strong antiangiogenic activity turned out to be useful for the treatment of multiple myeloma [3]. Investigating the efficacy of approved or discarded drugs for new indications using an approach called drug repositioning can in fact overcome some of the obstacles in drug discovery, such as the necessity to meet quality standards [6]. Reducing the failure rate drug repositioning also represents a reasonable chance to identify pharmacological tools against rare diseases and make personalized medicine more affordable by reducing the failure rate and therefore average cost of the drug discovery process [7], [8], [9].

### 1.2 Drugs, targets, and diseases

**Fig. 1:**
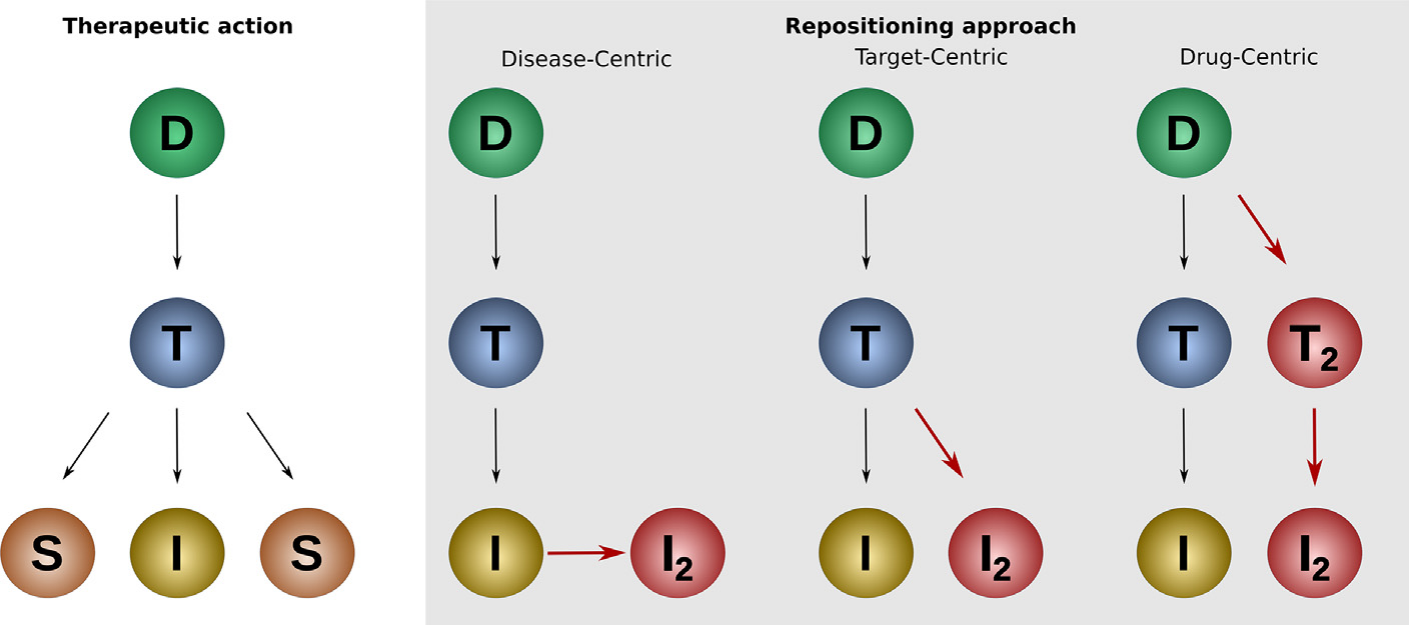
Different concepts behind drug repositioning. The relationships among drugs (D), targets (T) and indications (I) are represented for the different drug repositioning concepts. According to the receptor theory, the interaction of a small molecule drug (D) with one or more targets (T) has several biological effects, which can be useful for a therapeutic indication (I) or may produce undesired side effects (S). In disease-centric drug repositioning, a drug’s application is expanded from the original indication (I) to a closely related one (I2). In target-centric drug repositioning, the identification of a new indication (I2) is linked to a well established therapeutic target and in drug-centric drug repositioning, a newly identified drug target (T2) links the drug to a new indication (I2).

Like the above-mentioned cases of sildenafil and thalidomide, many drug repositioning stories derive from serendipitous or *a posteriori* observations. However, a systematic and rational strategy to link a known drug to a new indication is necessary to fully exploit the advantages of drug repositioning. Fig. 1 shows a simplified classification of different rational repositioning approaches. For all of these approaches, a functionally altered protein target plays a key role in the disease and a drug treats the disease by inhibiting or activating the target. Thus, drug repositioning can act on each of these three levels: disease, target, or drug. Focusing on the drug/disease relationship is the most direct way to repurpose a molecule since it is driven by the hypothesis that a drug’s use can be expanded from the original to a closely related indication. For instance, the tyrosine kinase inhibitor nilotinib was originally approved for the treatment of imatinib-resistant chronic myelogenous leukemia [10]. A few years later, Novartis proposed the repositioning of nilotinib to treat gastrointestinal stromal tumors. Disease-centric repositioning, as we define it, consists of the re-profiling of drugs among different types of a disease, such as two types of cancer. The underlying assumption for disease-centric repositioning is that different types of a disease share similar guiding principles. In the case of cancer, these guiding principles are summarized in the hallmarks of cancer [11]. Despite such commonalities, even closely related indications can have crucial differences that result in the failure of repositioning. For example, Novartis’ efforts to expand nilotinib to treat gastrointestinal stromal tumors were abandoned after a phase III trial found that the drug was not advisable to use for this indication [12]. Complementary to a disease-centric approach, target-centric repositioning builds on a novel link between a new indication and an established target. For example, the tyrosine-protein kinase ABL has recently been suggested as a novel target in Parkinson’s disease [13]. Hence, its inhibitors, such as nilotinib, might be effective against this syndrome [14]. This indication shift from cancer to neurodegeneration is driven by the target tyrosine-protein kinase ABL and represents a case of target-centric repositioning. Lastly, drug-centric repositioning occurs when a novel target connected to a certain indication is predicted for a given drug, as shown in Fig. 1. For example, valproic acid is for bipolar disorder and seizures because of its ability to bind to the mitochondrial enzymes succinate-semialdehyde dehydrogenase (ALDH5A1) and 4-aminobutyrate aminotransferase (ABAT). Valproic acid, however, does have an off-target interaction with the histone deacetylase 2 (HDAC2), a protein that plays a role in many types of cancers. It has been hypothesized that valproic acid induces differentiation, growth arrest, and apoptosis in cancer cells, leading to its repositioning to the treatment of neoplastic conditions such as familial adenomatous polyposis [15].

### 1.3 Drug-target interaction prediction in drug repositioning

A precise characterization of drug-target interactions allows for the generation of novel rational repositioning hypotheses following the drug-centric approach. Experimental identification of binding interactions can be challenging and expensive. Therefore, computational techniques for drug-target interaction prediction have gained a lot of attention in rational drug repositioning. Computational approaches can generally be divided into ligand-based, target-based, and machine learning-based approaches [16]. Ligand-based methods predict the binding affinity of ligands by comparing the candidate ligand with compounds that are known to be active against the therapeutic protein target. The performance of ligand-based approaches, such as Quantitative Structure–Activity Relationship (QSAR) and pharmacophore modeling, depends on the number of ligands known to be active against the target [17]. Target-based approaches, such as docking and binding-site similarity, are powerful tools for the identification of new repositioning cases. However, their performance is limited due to the scarce availability of target structures, as in the case of G-protein-coupled receptors (GPCRs). [17], [18]. Machine learning approaches predict novel drug-target pairs by identifying similarities among both compounds and targets. These approaches are generally classified into feature vector-based machine learning and similarity-based machine learning. Similarity-based machine learning methods can be further grouped into three categories: Kernel-based approaches, matrix factorization-based approaches, and network-based approaches [19]. Compared to the time consuming docking and information-demanding QSAR approach, machine learning methods can be faster and more efficient [20]. Nonetheless, some limitations to the machine learning approaches arise from the databases they commonly use, which sometimes miss important aspects of drug-target interactions, such as their dose-dependence and quantitative affinities [21].

### 1.4 Structure-based drug-target interaction prediction for drug repositioning

Several techniques are applied to predict drug-target interactions. These techniques commonly utilize structural information of the active or drug-binding site of the target to infer novel connections between drugs and targets. A study by Haupt et al. has shown that the binding of a drug to multiple different targets correlates with the binding site similarity of these targets. This suggests that there is a role for structural binding site analyses in drug repositioning [22]. All of the above-mentioned techniques have been successfully applied in drug repositioning to predict new therapeutic candidates. For example, Li et al. used a docking-based approach to find novel targets for existing drugs by computationally screening the whole druggable proteome. They validated nilotinib as a potent inhibitor of mitogen-activated protein kinase 14 (MAPK14), which adds an anti-inflammatory potential to nilotinib’s effects [23]. A similar strategy was used to identify new drugs against multi-drug resistant (MDR) and extensively drug resistant (XDR) tuberculosis. Based on the structural and interaction similarity between the original target catechol o-methyltransferase (COMT) and the new target inhibin alpha chain (INHA), the combination of anti-Parkinson’s Disease drug tolcapone with the drug entacapone (a levodopa anti-Parkinson’s Disease enhancer) has been predicted to be effective for the treatment of MDR and XDR tuberculosis [24]. Docking scores have also been fused with other structural information using data integration techniques. For example, the “train, match, fit, and streamline” (TMFS) method combines docking scores, ligand and receptor topology descriptor scores, and ligand-target interaction points to predict potential new drug-target interactions and provide structural insight into their mechanisms of action. Using this method, Dakshanamurthy et al. identified and validated two novel drug-target interactions: mebendazole-vascular endothelial growth factor receptor 2 (VEGFR2) and celecoxib-cadherin-11 (CDH11) [25]. Furthermore, several structure-based non-docking approaches found an extensive application in drug repositioning in order to overcome inefficiency and inaccuracy of docking. For instance, using information about the active-like state of the serotonin receptor 5-HT2C in complex with ergotamine and the inactive-like state of the same receptor in complex with ritanserin, Peng et al. predicted the binding of ergotamine to the delta-opioid receptor [26]. In another non-docking structure-based approach, Salentin et al. used interaction pattern comparison to identify novel repositioning candidates against the cancer target heat shock protein beta-1 (Hsp27). While analyzing the interaction patterns of the Hsp27 inhibitor brivudine, they found approved anti-malaria drug amodiaquine to be a promising anti-cancer agent [27]. Although many successful cases have proven, structure-based drug repositioning is limited by the little quantity of available structural information, particularly concerning certain classes of drug targets such as GPCRs.

### 1.5 Pros and cons of disease-, target-, and drug-centric repositioning

At first glance, disease-centric repositioning may appear faster and more linear than target- and drug-centric repositioning. A disease-centric repositioning hypothesis is based on a direct connection between the drug and its indication, therefore it allegedly avoiding a deeper understanding of the physicochemical interactions between drug and therapeutic targets. However, if similar diseases were always directly connected, one cancer drug would cure all other forms of cancer. Instead, disease-centric approaches require a detailed understanding of the disease phenotype and the underlying molecular processes in order to seek novel indications. Furthermore, disease-centric approaches may be affected by patents as the repositioning candidate and the corresponding old indication are usually protected by patent claims. Hence, the commercial exploitation of a disease-centric repositioning needs to be closely coordinated with the related patent claims. Systematic approaches to disease-centric repositioning typically define numeric similarities of diseases. These approaches include comprehensive and computational comparisons of disease phenotypes and drug side effects [28], [29] as well as comparisons of gene expression profiles [30]. In contrast to disease-centric approaches, target-centric repositioning approaches only search drugs where the old and new indications differ more clearly from one another. Therefore, it becomes less likely that the new indication is already covered by patents for the drug. However, a novel link from the target to a new indication is a rare finding. Consequently, these approaches are limited by the technologies available to uncover new target-disease associations. In addition to screening methods such as deep sequencing, micro-arrays and RNAi, which can provide clues to candidate targets, the target-centric approach requires a deep understanding of the molecular relation between the target and the disease. Drug-centric repositioning, on the other hand, can be considered the least direct approach because the drug is only linked to a novel indication via the discovery of a target that is already established for this indication. The best-known structure-based techniques for drug-centric repositioning are: molecular docking to screen single compounds against a library of protein structures [31], [32], [33], [34], [35], [36], [37]; pharmacophore modelling algorithm to screen protein–ligand 3D pharmacophoric features describing the ligand’s binding [38]; and protein–ligand interaction profile similarity approaches, which compare interaction patterns in the form of numerical fingerprints to study binding mode similarities of drugs and identify novel targets for the repositioning candidates [39], [40], [41]. All of the above techniques have been proven to be effective tools to illuminate new drug repositioning opportunities. However, the availability of data is a major limitation. Drug-centric approaches focus on the drug to be repurposed to another target/disease. Therefore, a crystallized structure describing the binding mode of the drug to its original targets is required. It is only possible to perform a screening to search for similar characteristics in other structures if this crystallized structural information is available. Since each repositioning approach has advantages and disadvantages, we performed a retrospective analysis to examine their distribution to the real cases of successful drug repositioning and to study the role of drug-target interaction prediction in drug repositioning.

## 2 Results

**Fig. 2:**
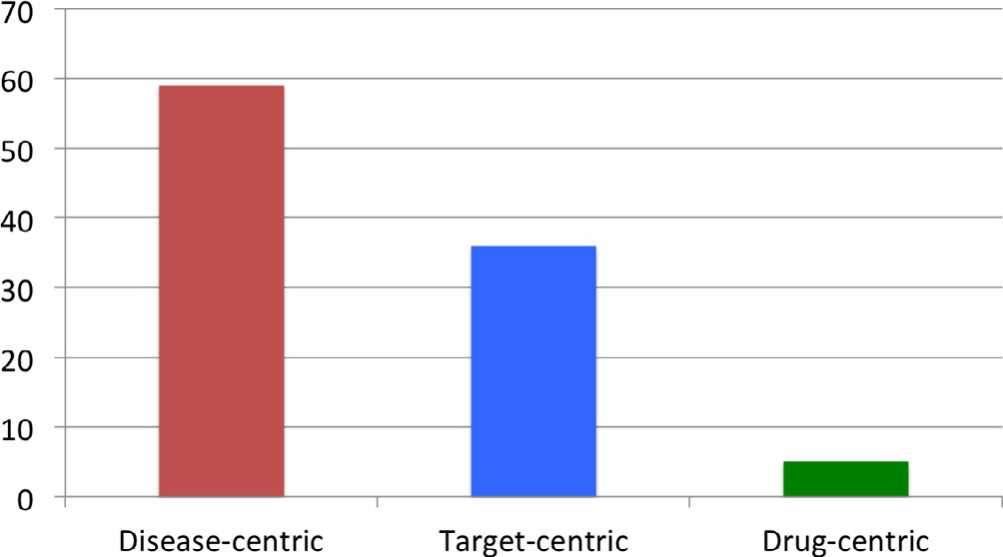
Summary of drug classifications. The bar chart shows the percentage of different typologies of repositioning approaches according to our classification. More than half of the analyzed cases (59%) were labeled as disease-centric repositioning cases, a third of the drugs (36%) were assigned to target-centric repositioning, while only a small group (6%) of cases were classified as drug-centric.

Which of the three approaches dominates the field of drug repositioning? Are drug-target interaction predictions a driving force of drug repositioning? To address these questions, we analyzed all of the repositioned small molecule drugs that are active against a protein target and present in the Repurposed Drug Database (RDD, http://www.drugrepurposingportal.com/repurposed-drug-database.php). We performed a classification of repurposed drugs according to the criteria specified in the “Methods” section. With this method, we determined the number of repositioning cases that can be assigned to the drug-centric approach. It should be noted that other classification criteria could be applied to shed light on different characteristics, which would generate different results from the same data set. It is also important to specify that the database does not contain any temporal information on the repositioning approaches. The classification results are summarized in Fig. 2.

### 2.1 Current drug repositioning set contains 128 known cases of small molecule drugs

The merging of the RDD with the Molecular Drug Targets (MDT) data has led to a compiled data set comprised of 196 drug repositioning cases, 263 unique targets and 333 unique indications. After removing the cases with non-small molecule drugs or non-protein targets, 128 repositioning cases constituted the starting point for our classification. A list of these cases is provided in Annex I.

### 2.2 The majority of repositioning cases (59%) was discovered via a disease-centric approach

**Fig. 3:**
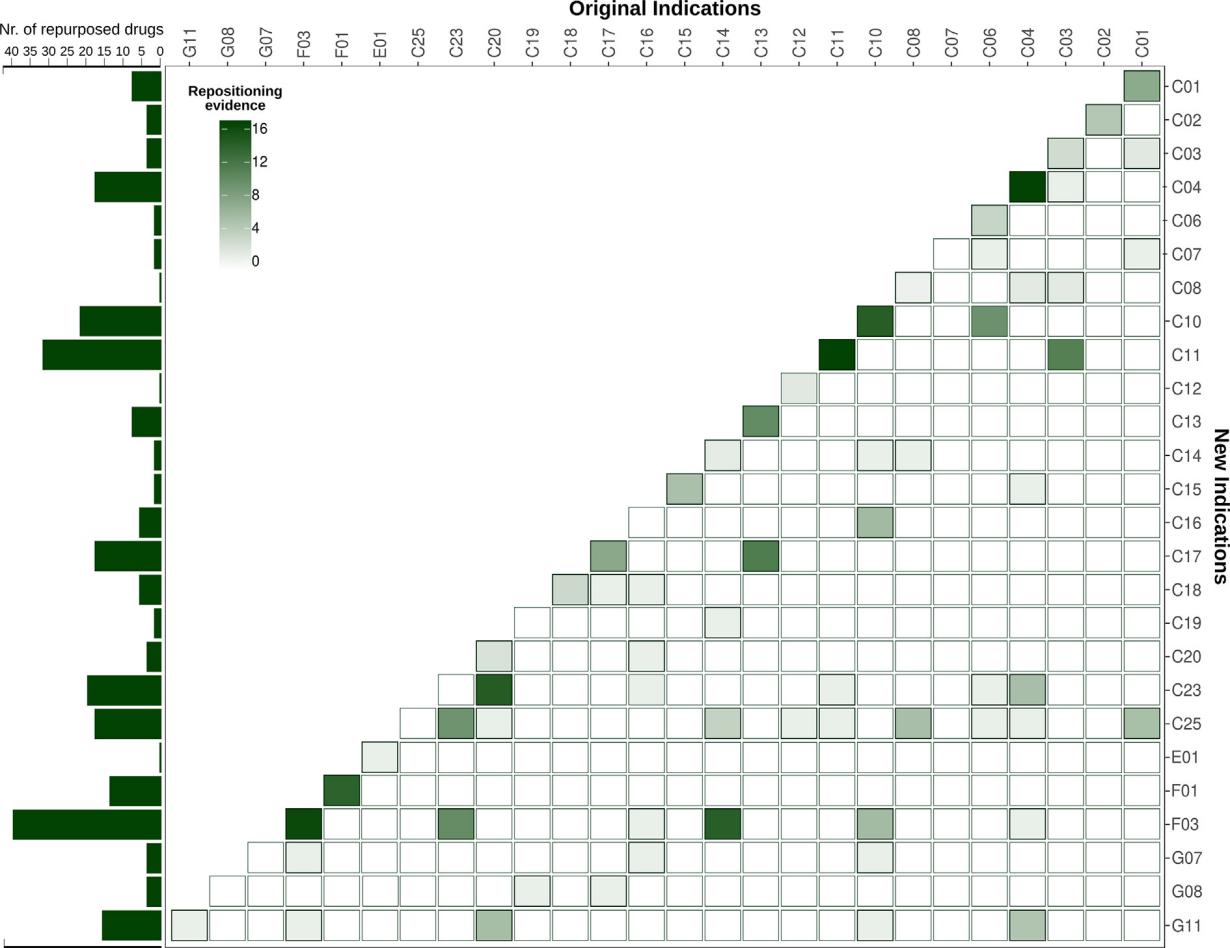
Frequency of repositioning cases among indication pairs. The figure shows the frequency of repositioning cases for each pair of primary and secondary indications. The disease classes are plotted on both axes and the number of repurposed drugs from one disease class to another is expressed by the color intensity of the square representing the respective disease class pair. The darkest squares lay on the central diagonal, showing that the majority of successful repositioning cases was discovered within the same disease class. On the left side of the plot, the number of repositioned drugs is displayed for the new indication.

To identify and characterize diseases that are susceptible to drug repositioning, we first determined the number of repurposed drugs for each type of disease (Fig. 3). With this in mind, diseases were distinguished by the root Medical Subject Headings (MeSH) term key. This key is a comprehensive and controlled vocabulary that provides a consistent way to retrieve information that may be described by variable terminology, thus facilitating indexing and searching. The MeSH vocabulary is organized into groups, of which one group is diseases (group C). The most common MeSH disease categories in the RDD are various neoplasms, immune system diseases, pathological signs and symptoms (clinical manifestations that can be either objective when observed by a physician, or subjective when perceived by the patient) and nervous system diseases. It is something worth nothing that repositioning cases not only exist in MeSH group C (diseases) but also in groups E01 (Diagnosis), F02 (Psychological Phenomena and Processes), F03 (Mental Disorders), G08 (Reproductive and Urinary Physiological Phenomena), and G11 (Musculoskeletal and Neural Physiological Phenomena). The analysis of the “original indication – secondary indication” pairs for the small molecule drugs (see Fig. 3) revealed that the most interesting repositioning cases are combinations of bacterial infections (C01) and parasitic diseases (C02 and C03) with other types of diseases. In these cases, the repositioning occurred either to a homolog protein with conservation of the function or to a completely different protein target. The antimycotic drug ketoconazole is an example of repositioning to a homologous protein which has been repositioned from a fungal target to the human homolog (Cytochrome P450) to treat cyclosporine-induced nephrotoxicity. Doxycycline, on the other hand, is an example of repositioning to a distinct target as it has been repurposed from an inhibitor of bacterial 30S ribosomal proteins S4 and S9 to an inhibitor of human metalloproteinase to treat stomatognathic disease. However, such repositioning cases are rare. Strikingly, the main diagonal of the ’original indication – secondary indication’ heatmap (Fig. 3) is the most populated, meaning that most drugs were repositioned within the same disease class. In total, 76 out of the 128 cases belong to the disease-centric repositionings. Most drugs (16 drugs) were repositioned from one type of neoplasm to another (C04–C04). For instance, the kinase inhibitor nilotinib has been repurposed from the treatment of Philadelphia chromosome positive chronic myelogenous leukemia to the treatment of gastrointestinal stromal tumors (Table 1). The repositioning within immune system diseases (C20-C20) is also very common with a total of 13 cases. For example, the steroid beclomethasone has been repositioned from the treatment of rhinitis to treat intestinal graft-versus-host disease (Table 1). Among nervous system diseases (C10-C10), eight repositioning cases were detected, including the repositioning of intravenous midazolam hydrochloride from its use as a preoperative sedation to being used against epileptic seizure activity (Table 1). Five cases were identified within “pathological conditions, signs, and symptoms” (C23-C23). In one of these cases, the repurposing of aminocaproic acid from enhancing hemostasis to topical treatment of traumatic hyphema of the eye (Table 1). Based on phenotypical and handling similarities, the groups C10, C23, and F03 (mental disorders) could even be combined to a large group of brain related diseases and perception modification. This would form the most frequent disease category in disease-centric drug repositioning. The overall high proportion of disease-centric repositioning cases could be explained by the existence of therapeutic targets that play a key role in the treatment of multiple similar diseases.

**Table 1:** Disease-centric repositioning cases. The 76 disease-centric repositioning cases grouped by indication category according to the MeSH tree classification. Since the original and secondary therapeutic indication fall within the same MeSH category, no further analyses on the targets were carried out. The MeSH indication names are ordered by the quantity of repositioned drugs.

N	MeSH category	Drug names	Number of drugs			
1	Neoplasms	Alitretinoin, Arsenic trioxide, Clofarabine, Daunorubicin liposomal, Doxorubicin, Erlotinib hydrochloride, Floxuridine, Idarubicin, Lapatinib, Nilotinib, Paclitaxel, Paclitaxel aqueous gel, Paclitaxel protein-bound particles for injection suspension, Pazopanib, Sorafenib, Toremifene	16			
2	Immune System Diseases	Azathioprine, Beclomethasone 17,21-diproprionate, Fludarabine phosphate, Leflunomide, Lenalidomide, Mesalamine, Mycophenolate mofetil, Nabumetone, Nevirapine, Pentostatin, Pralatrexate, Thalidomide, Vorinostat	13			
3	Nervous System Diseases	Apomorphine, Clonazepam, Gabapentin, Galantamine, Midazolam HCl, Memantine, Riluzole, Tetrabenazine	8			
4	Bacterial infections and Mycoses	Aztreonam, Clindamycin, Doripenem, Levofloxacin, Rifabutin	5			
5	Digestive System Diseases	Fluorouracil, Nitazoxanide, Nitisinone, Synthetic human secretin, Ursodiol,	5			
6	Mental Disorders	Aripiprazole, Atomoxetine hydrochloride, Fluoxetine, Milnacipran, Pramipexole	5			
7	Pathological Conditions, Signs and Symptoms	Aminocaproic acid, Bupivacaine, Medroxyprogesterone acetate, Midazolam nasal spray, Tramadol hydrochloride	5			
8	Respiratory Tract Diseases	Ambrisentan, Bosentan, Mifepristone, Nitric oxide, Tiotropium bromide	5			
9	Virus Diseases	Disoproxil fumarate, Ribavirin, Tenofovir	3			
10	Cardiovascular Diseases	Bethanidine Sulfate, Nitroprusside	2			
11	Female Urogenital Diseases and Pregnancy Complications	Progesterone, Testosterone propionate	2			
12	Hemic and Lymphatic Diseases	Anagrelide, Decitabine	2			
13	Behavior and Behavior Mechanisms	Bupropion	1			
14	Diagnosis	Synthetic porcine secretin	1			
15	Eye Diseases	Brimonidine	1			
16	Musculoskeletal and Neural Physiological Phenomena	Mepivacaine	1			
17	Nutritional and Metabolic Diseases	Miglustat	1			
18	Parasitic Diseases	Praziquantel	1			

### 2.3 36% of the repositioning cases fall into the target-centric category

For the 52 remaining repositoning cases (128 total minus 76 disease-centric cases) the drug targets were linked to the original and secondary indications using data mining and literature information. If the targets were the same for both indications or showed a protein sequence identity of at least 30% [42], the drug repositioning was classified as target-centric. There were 5 target-centric repositioning cases based on the binding of the drug to two homologous targets with the same function (orthologs) (see Table 2). The calculated sequence identities of the protein targets were 49% for ketokonazole, 57% for eflornithine, 60% for dapsone, 65% for atovaquone and 66% for trimetrexate. These values are much higher than the herein defined 30% similarity threshold. In total, 45 drug repositioning cases were classified as target-centric (Table 2). An example is chlorpromazine, whose interaction with the serotonin receptor HTR2A is involved in both the antiemetic/antihistamine indication (pathological conditions, signs and symptoms) and the non-sedating tranquillizer action (mental disorder) (Table 2). As an inhibitor of cyclooxygenase-2, the non-steroidal anti-inflammatory drug celecoxib was originally approved for the treatment of osteoarthritis and adult rheumatoid arthritis (immune system diseases). It has subsequently been repurposed to familial adenomatous polyposis (congenital, hereditary and neonatal diseases) (Table 2).

**Table 2:** Target-centric repositioning cases. Disease (MeSH category) and protein target (gene name or Uniprot ID) for both the primary and the secondary indication are shown. For 40 cases of target-centric repositioning, the target UniprotIDs are identical for original and secondary indication. Five drugs have been repurposed to/from a non–human ortholog target with a sequence similarity higher than 30%. The drugs are listed in alphabetical order. The references of target-disease associations retrieved from PubMed are given in the respective cells.

N	Drug name	Original Indication		Secondary Indication						
		MeSH Category	Gene Target	MeSH Category	Gene Target					
1	Adenosine	Congenital, Hereditary and Neonatal Diseases	ADORA1/2A/2B/3	Nervous System Diseases	ADORA1/2A/2B/3					
2	Albuterol	Respiratory Tract Diseases	ADRB2	Pathological Conditions, Signs and Symptoms	ADRB2					
3	Alfetanil	Musculoskeletal and Neural Physiological Phenomena	OPRM1	Nervous System Diseases	OPRM1					
4	Alprostadil	Mental Disorder	PTGER1/PTGER2	Cardiovascular Diseases	PTGER1/PTGER2					
5	Amiloride	Nutritional and Metabolic Diseases	SCNN1A	Congenital, Hereditary and Neonatal Diseases	SCNN1A					
6	Atovaquone	Pneumonia	Cytochrome b *(Pneumocystis carinii)*	Toxoplasma gondii encephalitis	Cytochrome b *(Toxoplasma gondii)*					
7	Azacitidine	Hemic and Lymphatic Diseases	DNMT1/3A	Neoplasms	DNMT1/3A					
8	Buprenorphine	Eye Diseases	OPRK1,OPRM1,OPRD1	Mental Disorder	OPRK1,OPRM1,OPRD1					
9	Capsaicin	Pathological Conditions, Signs and Symptoms	TRPV1	Cardiovascular Diseases	TRPV1					
10	Celecoxib	Immune System Diseases	PTGS2	Congenital, Hereditary and Neonatal Diseases	PTGS2					
11	Chlorpromazine	Pathological Conditions, Signs and Symptoms	DRD2-4,HTR2A/2C,HRH1/4	Mental Disorder	HTR2A					
12	Dapsone	Dermatitis herpetiformis	Dihydropteroate synthase *(Mycobacterium leprae)*	Toxoplasmosis	Dihydropteroate synthase *(Toxoplasma gondii)*					
13	Desmethylmifepristone	Respiratory Tract Diseases	NR3C1	Endocrine System Diseases	NR3C1					
14	Dexamethasone	Eye Diseases	NR3C1	Immune System Disorders	NR3C1					
15	Difluprednate	Pathological Conditions, Signs and Symptoms	NR3C1	Eye Diseases	NR3C1					
16	Dihydrodigitoxin	Cardiovascular Diseases	ATP1A1-4	Endocrine System Diseases	ATP1A and more [43]					
17	Dimethylstilberstrol	Female urogenital Diseases and Pregnancy Complications	KEAP1	Skin and Connective Tissue Diseases	KEAP1					
18	Duloxetine	Mental Disorder	SLC6A,SLC6A4	Pathological Conditions, Signs and Symptoms	SLC6A4					
19	Eflornithine	African trypanosomiasis	Ornithine decarboxylate *(Trypanosoma Brucei)*	Pneumocystis carinii pneumonia	Ornithine decarboxylate *(Pneumocystis carinii)*					
20	Epoprostenol Sodium	Respiratory Tract Diseases	PTGIR,PTGER1	Pathological Conditions, Signs and Symptoms	PTGIR,PTGER1					
21	Ethinyl Estradiol	Respiratory Tract Diseases	ESR1	Skin and Connective Tissue Diseases	ESR1					
22	Everolimus	Immune System Diseases	FKBP1A	Digestive System Diseases	FKBP1A					
23	Finasteride	Male Urogenital Diseases	SRD5A1/2	Pathological Conditions, Signs and Symptoms	SRD5A1/2					
24	Glycopyrrolate Bromide	Digestive System Diseases	CHRM1-5	Stomatognathic Diseases	CHRM1-5					
25	Guanethidine	Cardiovascular Diseases	SLC6A2	Nervous System Diseases	SLC6A2					
26	Guanfacine	Mental Disorder	ADRA2A/2B/2C	Congenital, Hereditary and Neonatal Diseases	ADRA2A/2B/2C					
27	Histamine	Immune System Diseases	HRH1	Neoplasms	HRH1					
28	Iloprost	Respiratory Tract Diseases	PTGIR	Cardiovascular Diseases	PTGIR					
29	Ketokonazole	Fungal infection	Cytochrome P450 *(Candida albicans)*	Nephrotoxicity induced by cyclosporine	Cytochrome P450 *(Homo sapiens)*					
30	Levomilnacipran	Mental Disorder	SLC6A2/4,	Nervous System Diseases	SLC6A2					
31	Mecamylamine Hydrochloride	Cardiovascular Diseases	CHRNA3/B4	Mental Disorder	CHRNA3/B4					
32	Metyrosine	Neoplasms	TH	Mental Disorder	TH					
33	Minoxidil	Cardiovascular Diseases	ABCC9	Pathological Conditions, Signs and Symptoms	ABCC9					
34	Misoprostol	Digestive System Diseases	PTGER3	Pathological Conditions, Signs and Symptoms	PTGER3					
35	Oxandrolone	Physiological Phenomena	AR	Congenital, Hereditary and Neonatal Diseases	AR					
36	Phentolamine	Cardiovascular Diseases	ADRA1A/1B/1D/2A/2B/2C	Mental Disorder	ADRA1A/1B/1D/2A/2B/2C					
37	Propranolol	Pathological Conditions, Signs and Symptoms	ADRB1-3	Neoplasms	ADRB1-3					
38	Raloxifene	Nutritional and Metabolic Diseases	ESR1/2	Skin and Connective Tissue Diseases	ESR1/2					
39	Ropinirole	Cardiovascular Diseases	DRD2-4	Mental Disorder	DRD2-4					
40	Sibutramine	Mental Disorder	SLC6A2-4	Physiological Phenomena	SLC6A2-4					
41	Sildenafil	Pathological Conditions, Signs and Symptoms	PDE5A	Mental Disorder	PDE5A					
42	Tadalafil	Cardiovascular Diseases	PDE5A	Mental Disorder	PDE5A					
43	Tranexamic Acid	Pathological Conditions, Signs and Symptoms	PLG	Immune System Disorders	PLG					
44	Tretinoin	Neoplasms	RARA/B/G	Musculoskeletal and Neural Physiological Phenomena	RARA/B/G					
45	Trimetrexate	Pneumonia	Dihydropholate reductase *(Pneumocystis carinii)*	Metastatic carcinoma of the head and neck	Dihydropholate reductase *(Homo sapiens)*

**Table 3:** Drug-centric repositioning cases. For each drug-centric case, therapeutic indications (MeSH category) and protein targets (gene name or Uniprot ID (*:Non–human, (+):multiple subunits)) for both the original and the secondary indications are shown. According to our definition of drug-centric repositioning, these seven cases must have a different MeSH code and protein target for the primary and secondary indication. The seven cases are most interesting for the application of drug-target interaction prediction techniques because they have the highest target and indication diversity. The drugs are listed in alphabetical order. The references of target-disease associations retrieved from PubMed are given in the respective cells.

N	Drug name	Original Indication		Secondary Indication				
		MeSH Category	Gene Target	Category	Gene Target			
1	Allopurinol	Neoplasm	Xanthine dehydrogenase/oxidase *(Homo sapiens)*	Parasitic Diseases	Hypoxanthine phosphoribosyltransferase *(Trypanosoma Cruzi**[*44*]**)*			
2	Doxycycline	Bacterial Infection and Mycoses	*rpsD,*rpsI	Stomatognathic Diseases	MMP1/7/8/13			
3	Lidocaine	Musculoskeletal and Neural Physiological Phenomena	SCN1A/2A/3A/4A/5A/7A/8A/9A/10A/11A	Immune System Disorders	various/not specified (cytokines release) [45]			
4	Mazindol	Stomatognathic Diseases	SLC6A2-4	Congenital, Hereditary and Neonatal Diseases	various/not specified (growth hormone release) [46]			
5	Topiramate	Nervous System Diseases	GABR(+),GRIK1-5,GRIA1-4,SCN(+)	Stomatognathic Diseases	CA2/4			
6	Valproic acid	Nervous System Diseases	ALDH5A1,ABAT	Congenital, Hereditary and Neonatal Diseases	HDAC2			
7	Zidovudine	Neoplasms	HIV1 Reverse transcriptase [45]	Immune System Disorders	Human DNA polymerase [47]			

### 2.4 5% of the repositioning cases were classified as drug-centric

The remaining seven cases that could not be assigned to either disease- or target-centric repositioning were classified as drug-centric repositioning. In these cases, the primary and secondary indications are linked to distinct protein targets. Valproic acid is one instance of this. Valproic acid was originally developed to treat episodes of bipolar disorder and seizures (nervous system diseases) by hitting the mitochondrial enzymes succinate-semialdehyde dehydrogenase (ALDH5A1) and 4-aminobutyrate aminotransferase (ABAT). Since then it has been repurposed for the treatment of familial adenomatous polyposis (congenital, hereditary and neonatal diseases) based on its interaction with histone deacetylase 2 (HDAC2), as shown in Table 3. The drug allopurinol has been repurposed from a human protein target (Xanthine dehydrogenase/oxidase) to a parasitic target (Hypoxanthine phosphoribosyltransferase of Trypanosoma Cruzi) with a low protein sequence identity (6%).

### 2.5 Indication-target-drug network analysis

Three different network graphs, one for each repositioning approach (disease-, target-, and drug-centric), were built to illustrate and analyze the relationship between the identified repositioning cases. The results of the analysis are shown in Table 4. First, we analyzed the general structure of the networks (Table 4, General network information). The networks differ significantly in size due to the higher number of disease- and target-centric cases in comparison to the number of drug-centric repositioning cases. Interestingly, the disease- and drug-centric networks exhibit two main clusters, whereas the target-centric network features only one main cluster, comprising 84% of the nodes. Because disease descriptions varied, top-level MeSH categories were used as disease identifiers to integrate the different drug repositioning cases into a single network. It is important to know that, although the disease- and drug-centric networks both have two main clusters, the MeSH categories included in these clusters differ between the two repositioning approaches (disease-centric: C02-C20-C06-E01-C04 and C08-C14-F01-F03-C11-G11-C10-C23-C13, disconnected C03, C15, C18, and C01; drug-centric: C03-C04-C20-G11 and C10-C07-C16-C01). This shows that different approaches to drug repositioning allow the combination of different diseases. Secondly, we calculated the clustering coefficients for the drug, target, and the disease nodes in all three networks (Table 4, Nodes clustering). Clustering coefficients express how likely it is that the nodes form subsets that constitute an independent subgraph. The formation of such an independent subgraph is undesirable for drug repositioning because it excludes the possibility that a node is connected to a distinct part of the network. For instance, a drug might not be connected to a new target. Hence, a low clustering coefficient indicates that the respective node type (drug, target, or disease) plays a crucial role in the particular repositioning approach. For all three repositioning approaches, there are differences between the clustering coefficients of the drug, the target, and the disease nodes. Conspicuously for all three node types, the type of repositioning approach for which the lowest clustering coefficient was calculated matches the node type. This shows that target-nodes play a more important role in target-centric repositioning than in disease- or drug-centric repositioning. Moreover, the average clustering coefficient of the drug nodes decreases from the disease- over the target- to the drug-centric network. This means that in drug-centric repositioning, drugs are less prone to cluster around a single indication than in target- or disease-centric repositioning. Furthermore, none of the protein targets in the drug-centric network are connected to multiple drugs at the same time. Thirdly, we evaluated the small-world network properties of the graphs (Table 4, Small-world network properties). Small-world network is a graph-theory concept that is applied to measure how likely it is that the neighbors of one node are also neighbors of each other. In drug repositioning it is expected that a drug is highly unspecific or that the novelty of a repositioning case is low if the clustering of the nodes is too high. We calculated the small-world properties as the product of transitivity and the number of nodes divided by the effective diameter. The higher the coefficient, the higher the small-world network properties. The lowest and highest small-world network properties were identified for the drug-centric network and the disease-centric network respectively. The low small-world properties of the drug-centric network indicate that the drugs in this network are more likely to engage in distant connections.

**Table 4:** Analysis of indication-target-drug networks. Three different networks were generated, one for each repositioning approach (disease-centric, target-centric, and drug-centric). General network information, average clustering coefficients of the different node types, and small-world network properties are listed for each repositioning approach. For all three node types, the lowest clustering coefficient was found for the type of repositioning approach that matches the type of the nodes (lowest coefficient is highlighted in bold for each node type), which demonstrates the importance of the respective node type in drug repositioning. Small-world network properties (estimated as the product of transitivity and the number of nodes divided by the effective diameter) are highest for the disease-centric network (5.90) and lowest for the drug-centric network (4.01).

	Disease-centric	Target-centric	Drug-centric	
*General network information*				
Number of edges	466	327	84	
Number of nodes	232	147	51	
Percentage nodes biggest component	45.26	83.67	52.94	
Percentage edges biggest component	42.92	90.83	54.76	
				
*Nodes clustering*				
Average drug nodes clustering coefficient	0.72	0.57	**0.25**	
Average disease nodes clustering coefficient	**0.28**	0.52	0.50	
Average target node clustering coefficient	0.78	**0.69**	1.00	
				
*Small-world network properties*				
Effective diameter of the biggest component	5.16	4.99	3.43	
Graph transitivity	0.13	0.17	0.27	
$\mathrm{transitivity}\cdot \frac{\mathrm{number}\;\mathrm{of}\;\mathrm{nodes}}{\mathrm{effective}\;\mathrm{diameter}}$	5.90	5.13	4.01	

### 2.6 None of the drug-target pairs from the drug-centric repositioning cases had sufficient structural data for structure-based drug-target interaction prediction

Finally, we wanted to use our classification system to assess the putative impact of structure-based drug-target interaction prediction on drug repositioning. Three dimensional information about the position of the drug inside the active site of a target can be extremely helpful to understand the drug’s binding behavior and to generate repositioning hypotheses. Such data can be generated by crystallography or ligand–protein docking, and may be analyzed via interaction-profile similarity approaches. Many drug repositioning pipelines use structure-based techniques to obtain and confirm molecular binding hypotheses. However, the impact of these approaches on the actual status of drug repositioning is not clear. Hence, after defining a list of drug-centric repositioning cases (the ones which could benefit most from structure-based techniques), we tried to understand to what extent structure-based techniques could be useful for drug repositioning. We screened the Protein Data Bank (PDB) [48] to check the availability of structural data for the seven repositioning cases classified as drug-centric. Interestingly, the structures of the drug in complex with both the original and the secondary target were not available for any of the drug-centric repositioning cases.

## 3 Discussion

### 3.1 More than a third of the cases do not fit the ’small molecule drug – protein target’ definition

We focused our analysis exclusively on small molecule drugs because they usually bind to a higher number of different targets and form more defined interactions with those targets. Moreover, we only considered drug targets that are proteins. Altogether, 68 out of 196 repositioning cases were excluded from the analysis as they did not fit into the “small molecule drug – protein target” scheme. Non-small molecule drugs were typically antibodies while non-protein targets included RNA, DNA and other non-protein biomolecules. Examples of repositioned therapeutic antibodies are infliximab and adalimumab, which are both used for Crohn’s disease and juvenile rheumatoid arthritis. The database also included therapeutic proteins such as somatropin, which is used to treat children with growth disorders and to induce ovulation in infertile women. An example of drugs that have a non-protein drug target is melphalan. Melphalan binds DNA and is applied in multiple myeloma as well as in metastatic melanoma. DNA is also the target of cladribine, which is used for the treatment of hairy cell leukemia and chronic lymphocytic leukemia.

### 3.2 Drug repositioning is mostly disease- and target-centric

The retrospective analysis of the repositioning cases present in the RDD database gave us an interesting picture of the current state of drug repositioning. Sixty percent of the repositioned drugs analyzed (76 cases out of 128) have been redirected to the same disease family. This tendency was particularly pronounced within two categories of therapeutic indications: neoplasms and immune system disorders (Fig. 3). These also have the highest number of repositioning cases in the database. Thirty percent of the analyzed drugs (45 out of 128) have been repurposed to a different indication but to the same protein target, as indicated by an identical Uniprot ID or high protein sequence identity. Only 5%, or seven cases, have been repositioned to a different disease and a different target. The situation described here seems to reflect a general trend in drug discovery. This situation is considered one of the reasons for the structural crisis in pharmaceutical R&D mentioned in the introduction: the current pharmaceutical R&D situation has been compared to a oil-drilling process, where the cheapest and easiest options with highest expected returns are exploited first and less attractive options are left behind [49]. In terms of our results, this could mean that certain disease classes and rapid repositioning approaches within the same disease and target family are prioritized, leaving a big pool of drug-target-disease connections virtually unexplored. In light of this, it might be crucial in the future to invest in drug repositioning techniques that focus on the fine characteristics of drugs, targets and diseases (drug-centric approaches), thereby overcoming the barriers defined by a disease category or target. A systematic and efficient repositioning approach that connects unrelated diseases and targets might benefit both the pharmaceutical R&D and the patients by increasing the profits and delivering novel therapeutic agents in a fast and cost-effective way.

### 3.3 Indication-target-drug network analysis confirms our classification

We analyzed drug-centric, target-centric and indication-centric networks, and demonstrated that they all have different structures and represent different approaches to drug repositioning. For the disease-, target-, and drug-centric networks, disease, target, and drug nodes played the most important role in drug repositioning, respectively, showing the validity of our analysis. In addition, we evaluated the differences in the small-world network properties between the three networks. Drug-centric approaches showed the lowest small-world network properties. Based on this result, we assume that using the drug-centric approach makes it possible to find drug repositioning cases of superior novelty and higher specificity compared to disease- and target-centric repositioning.

### 3.4 The role of drug-target interaction analyses in drug repositioning

Although computational drug repositioning has lately developed many strategies for predicting drug-target interactions, our analysis shows that most of the actual repositioning cases could be the result of a disease-centric or target-centric approach. As shown in Fig. 1, disease-centric drug repositioning can directly link a drug to a pathological condition with no need for assessing target similarity or analyzing drug-target interactions. Target-centric drug repositioning, on the other hand, requires a firm connection between target and indication. Platforms like Open Targets [50] and Beagle [51] facilitate the identification of such connections. However, the target-indication link is often not so direct and clear. Finally, the drug-centric repositioning cases are the only ones that could have really benefited from drug-target interaction prediction methods (ligand-based, structure-based and machine learning-based).

### 3.5 The limits and potential of structure-based drug repositioning as drug-centric approach

Structure-based drug repositioning techniques are examples of drug-centric approaches. They can be applied to infer new interactions between drugs, targets, and indications by considering information about the structure of the drugs, the targets, and their interactions. Although structure-based drug repositioning has great potential for the repurposing of known drugs to different targets and indications [52], [27], several limitations make it less easy to apply this approach in a relevant and systematic way. In fact, structural data for both the original drug and its target are required. Structural data may also be required for the potential new target and, possibly, its ligands. Lack of this information considerably limits the searching space for drug repositioning. Actually, there were no cases classified as drug-centric repositioning where both the original and the repositioned drug-target complex were available in the PDB. This confirms the barriers of structure-based drug repositioning. To tackle the scarce data availability, various techniques such as homology modeling and molecular docking can be used to predict the structure of a protein and its interaction with a query drug. However, generating reliable data requires considerable expertise and computational power.

### 3.6 Database selection and data availability

By the time we started this work (2016), the RDD was the only comprehensive drug repositioning database available. Later, other data sets of repurposed drugs were published. For instance a new gold standard database of successful and failed repositioning cases, repoDB, was released in 2017 [53]. Despite differences in size between the RDD and the repoDB, we have decided to present our analysis as a proof-of-concept for the present drug repositioning situation. We are currently unaware of previous work based on the RDD, but the database is manually curated and validated. We used the MDT database to retrieve drug-target connections. Although many others such as DrugBank were available for this scope. The MDT database is the result of a comprehensive and accurate annotation that considers several sources of targets for FDA approved drugs [54]. However, the combination of the RDD and the MDT has resulted in a data reduction that may have compromised the quality of our analysis.

### 3.7 Issues related to the classification process

The aim of this study was to assess the impact of new drug-target interactions on the analyzed drug repositioning cases. In fact, our work is a retrospective analysis based on the final results of different successful repositioning processes and does not take into account the specific techniques used to repurpose the respective drugs (i.e. ligand-based, target-based, network-based and machine-learning based). The assignment of specific methods or hypotheses to each case of repositioning would have required a tremendous manual effort and would not have added essential information to our analysis. In addition, many repositioning processes require the application of multiple techniques. This made it difficult to assign a clear and unambiguous classification. Furthermore, we assume that many repurposed drugs that we have assigned to disease-centric repositioning could also be a product of a target-centric or drug-centric approach. This assignment was based off of a similarity between the drugs’ original and secondary indication. For example, following target affinity experiments, the drug nilotinib, which we have classified as disease-centric repositioning, has been repurposed to treat gastrointestinal stromal tumors by affecting a different protein target [7]. Moreover, the classification process was carried out step by step, beginning with the cases where the indications had an identical MeSH code (disease-centric) and continuing with those where the target was identical (target-centric). If a case was labeled as disease-centric, no further examination of the target was performed, thereby possibly missing overlaps between the different drug repositioning categories. For the reasons given above, a deeper molecular understanding of drug repositioning databases could lead to a more detailed analysis and provide a clearer picture of the true state of repurposing.

## 4 Methods

The data presented above are the product of a retrospective analysis carried out on a list of repurposed drugs, their indications, and protein targets from the Repurposed Drug Database (RDD), a list of Molecular Drug Targets (MDT), and literature. The results are the product of a classification process explained in details below, consisting of a step-wise division of the drug repositioning cases into a same-disease group and a same-target group.

### 4.1 Identification of current drug repositioning cases

The RDD was downloaded from www.drugrepurposingportal.com in January 2017. The database contains 438 drugs that have been repurposed and provides information about the drugs with their original and new indications. This includes drugs that have been unsuccessful, approved, or experimental in connection with the original indication and have later been approved for another new indication. Given the above, it was necessary to integrate this data into another source providing information about the protein targets involved in both indications.

### 4.2 Retrieval of molecular drug targets data

The therapeutic targets of both launched and potential drugs are often poorly defined in the literature. Several databases provide data on drug-target interactions with different foci on the content. Examples of such databases are the Therapeutic Targets Database [55], Drugbank[56], SuperTarget [57], and the IUPHAR/BPS Guide [58]. Despite the variety of valuable online resources, it is still challenging to retrieve consistent and comprehensive data of drug targets with their molecular efficacy and therapeutic use, especially when mapping targets to specific genes and gene products. Santos et al. [54] presented a solution to the problem. They carried out a “comprehensive map of molecular drug targets” in which they curated 893 human and pathogen-derived biomolecules, which are targeted by 1578 FDA-approved drugs. Santos data set was downloaded in.php format in January 2017 and the list of MDT was retrieved from it. Importantly, Santos et al. specified that ’biomolecules that the drug may also bind to, or be metabolized by, but which are not known to be responsible for its therapeutic effect, are not defined as targets’. Moreover, the work defines a drug as any therapeutic agent, including not only small molecules, but also other biological agents that are used to enhance health.

### 4.3 Identification of targets involved in the repositioning cases

**Fig. 4:**
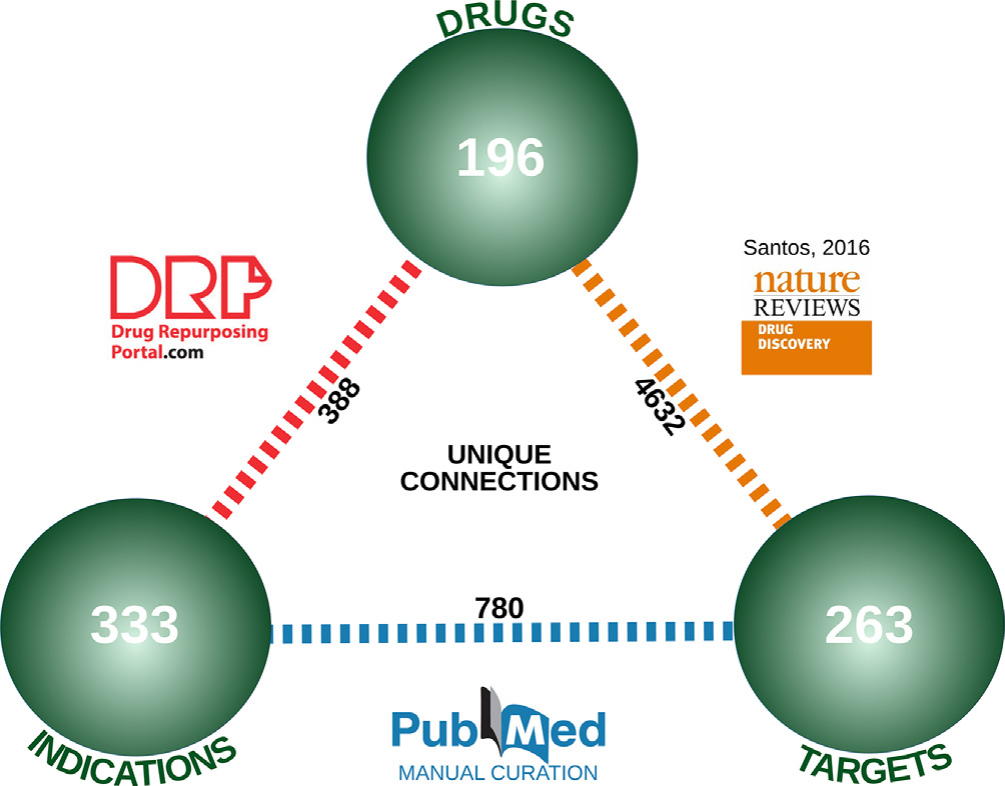
Collection and classification of known repositioning cases. Merging of the Repositioned Drug Database (RDD), containing 196 drugs and 333 indications linked through 388 connections, the Molecular Target Database from Santos (MTD), containing 4632 links between 196 drugs and 263 targets, and PubMed, which allowed us to find 780 different links between 263 targets and 333 indications.

The data in the RDD were merged with the data in the MDT based on the commonly known drug names. This resulted in a data set of 196 drugs, 263 unique targets, and 333 unique indications (see Fig. 4 or Annex I for more details). The data was merged in April 2017 using a Python 2.7 script and complemented with other biologically relevant databases in order to enrich the analysis (see Annex I). For some drugs, no protein target was stored in the MDT and a manual literature search was necessary.

### 4.4 Filtering of non-small molecules and non-protein targets

This study only considered proteins and small molecules as therapeutic targets and drugs, respectively. Cases differing from this definition were excluded. These cases included antibody drugs and biomolecular targets distinct from proteins (e.g enzymes, DNA, or unknown). Of the 196 drugs included in the merged RDD and MDT data set, 68 were removed using these filter criteria (see Annex II).

### 4.5 Identification of disease-centric repositioning cases

A repositioning case was considered as disease-centric if the repurposing was based on disease phenotype similarity. Several databases support the correct indexing of diseases: ICD-10 [59], the Elsevier Emtree [60], and the Medical Subject Headings (MeSH [61]. MeSH and Emtree are the most commonly used databases. MeSH is usually preferred due to free access, its extensive history notes, its large scope notes [62], and its terminology in nursing, veterinary medicine and dentistry. For each repositioning case, the MeSH tree root keys were assigned to the respective indications (see Table 5). The frequency of repurposed cases among the indications was calculated and visualized using the Matplotlib python library. The number of cases for each root MeSH key pair was plotted in R with the ggplot2 package (see Fig. 3). The number of targets was calculated for each indication pair with identical root MeSH key. References to the applicability of these targets was also collected. Drugs linked to the same MeSH key were classified as cases of disease-centric repositioning.

**Table 5:** Root MeSH tree keys for the disease groups with corresponding disease descriptions. MeSH category codes (left column) and common names (right column) of all diseases and conditions for which drug repositioning cases are recorded in the RDD.

MeSH Category	Disease group	MeSH Category	Disease group				
C01	Bacterial Infections and Mycoses	C16	Congenital, Hereditary, and Neonatal Diseases and Abnormalities				
C02	Virus Diseases	C17	Skin and Connective Tissue Diseases				
C03	Parasitic Diseases	C18	Nutritional and Metabolic Diseases				
C04	Neoplasms	C19	Endocrine System Diseases				
C06	Digestive System Diseases	C20	Immune System Diseases				
C07	Stomatognathic Diseases	C23	Pathological Conditions, Signs and Symptoms				
C08	Respiratory Tract Diseases	C25	Chemically-Induced Disorders				
C10	Nervous System Diseases	E01	Diagnosis				
C11	Eye Diseases	F01	Behaviour Mechanisms				
C12	Male Urogenital Diseases	F03	Mental Disorders				
C13	Female Urogenital Diseases and Pregnancy Complications	G07	Physiological processes				
C14	Cardiovascular Diseases	G08	Reproductive and Urinary Physiological Phenomena				
C15	Hemic and Lymphatic Diseases	G11	Musculoskeletal and Neural Physiological Phenomena				

### 4.6 Target assignment to original and secondary indication

In order to divide the targets into original and secondary indication we used literature evidence in PubMed (see Fig. 4). Target-indication connections were retrieved manually from literature by searching for direct, indirect, or generalized evidence. Direct evidence included the improvement of a condition upon the treatment with a certain drug due to the action of this drug on the therapeutic protein target. The correlation between a disease condition and a certain target activity was considered as indirect evidence. Generalized evidence included single reports of target-condition links without a strong correlation being detected. The validation of the above-described manual curation was done via text-mining using the ensemble biclustering algorithm (EBC), which allows to extract the connections from a natural text in a machine-processable form. The text-mining data set used in this work consists of two parts. Part I connects dependency paths to labels or “themes”. They were introduced in this data set to label what kind of interactions exist between two terms, e.g. whether a causal mutation has a role in pathogenesis or promotes progression of the disease. The second part of the data set contains information about drug-target-indication associations. To validate the manual PubMed curation and estimate how applicable text-mining is for this aim in general, target-indication associations in this data set were used. To make the text-mining data set compatible with the drug repositioning data set, UniProt IDs were turned into gene IDs using the UniProt API service and MeSH on demand was used to assign indication IDs to the textual description of diseases. Afterwards, all of the records containing genes of therapeutic protein targets for drug repositioning were checked for the presence of a gene ID – MeSH ID pair. To identify whether the gene encodes a drug target, Part I and Part II of the text-mining data set were linked and the entries in which the gene actually had a “drug target” label were selected. In this way, targets were again divided into original and secondary indication (see Annex III). The resulting distribution was compared to the manual distribution.

### 4.7 Identification of target-centric repositioning cases

A repositioning case was considered as target-centric if the same protein target is used in different pathological contexts. The repositioning cases that were not classified as disease-centric were analyzed to determine whether the drug acts on two different protein targets related to original and secondary indication. First, a repositioning case was marked as target-centric if the UniProt IDs of the therapeutic protein targets were the same for both indications. Furthermore, a repositioning case was considered as target-centric if the UniProt IDs were different but the targets were homologous proteins with the same function in different organisms (ortholog). Therefore, the protein sequence identity was evaluated using Clustal Omega alignment (https://www.ebi.ac.uk/Tools/msa/clustalo), where the number of similar and identical aligned amino acids was summarized and divided by the length of the alignment. According to the accuracy cutoff established by Rost et al. [42] protein targets showing a sequence identity higher than 30% were considered as homologous and the respective repositioning case was classified as target-centric.

### 4.8 Identification of drug-centric repositioning cases

A repositioning case was considered as drug-centric if it exploits the chemical properties of a drug. First, the cases that were neither classified as disease-centric nor target-centric were considered as potential drug-centric repositioning cases. Cases were also classified as drug-centric if the target associated with the secondary indication was not included in the comprehensive map but there was literature evidence for different therapeutic targets.

### 4.9 Structural data availability for drug-centric repositioning cases

Finally, we evaluated whether it would have been possible to identify the drug-centric repositioning cases using a structure-based approach. Therefore, the UniProt IDs of the protein targets were mapped to PDB IDs and the PDB was searched for all available structures describing the binding between the drugs and their corresponding targets (associated to both primary and secondary indication). Additionally, structures that describe the binding of the drugs to targets that are not related to the drug repositioning cases (not described in the MDT) were considered. These structures were also evaluated regarding their potential for structure-based drug repositioning.

### 4.10 Analysis of the drugs-targets-indications network

To investigate the drugs-targets-indications network, the Python Networkx and SNAP [63] modules were used. The graph structure was established for each of the graphs (indication-centric, target-centric, drug-centric). For the indication-centric graph, the nodes are indications as the top-level of MeSH category. For the target- and drug-centric graph, the nodes for the indications are based on the data presented in Tables 2 and 3. In all graphs, unique protein targets and unique drugs are illustrated as unique nodes. Edges represent drug-target, target-indication, and drug-indication associations, which were established via the above-described analyses. The effective diameter is the 90th percentile of the distribution of the shortest path lengths of a graph. Diameters were calculated using the SNAP module. Since small-network properties are higher when the transitivity of the graph is higher and the diameter of the graph is smaller, we estimated relative small-world properties based on the (1).

(1) $$\mathrm{transitivity}\cdot \frac{\mathrm{number}\;\mathrm{of}\;\mathrm{nodes}}{\mathrm{effective}\;\mathrm{diameter}}$$

## 5 Conclusion

Drug-target interaction prediction is an important part of most of the rational drug repositioning approaches. In fact, different biochemical, physical, and mathematical techniques have been designed and optimized to accurately infer links between ligands and proteins. In this work, we analyzed various successful drug-repositioning cases. Based on the similarity between old and new indications and old and new targets, we evaluated the actual impact of drug-target interaction prediction on these cases. By dividing all the cases falling within the definition “small molecule – protein target” (128) into disease-centric (with very similar indications), target-centric (with identical or orthologue targets), and drug-centric (with different targets in different indications) drug repositioning, we found that only 7 out of 128 cases would have required drug-target interaction prediction to rationally initiate drug repurposing. This unexpectedly small number could potentially be explained by the higher amount of information, time, and money required for drug-target interaction prediction compared to target- and disease-centric approaches. A more detailed analysis of the drug-target complexes present in the PDB revealed that there is currently not enough structural data available for any of the repositioning cases classified as drug-centric. Therefore it is impossible to identify new drug-target interactions using structure-based techniques such as interaction profile similarity. On the other hand, these results highlight the existence of a big unexplored niche for drug-target interaction prediction in drug repositioning. This great potential will increasingly be used since the techniques for detecting new links between ligands and protein targets, such as structure-based drug repositioning, are constantly evolving.

## Declaration of Competing Interest

The authors declare that they have no known competing financial interests or personal relationships that could have appeared to influence the work reported in this paper.
